# Emotional Intelligence and Mismatching Expressive and Verbal Messages: A Contribution to Detection of Deception

**DOI:** 10.1371/journal.pone.0092570

**Published:** 2014-03-21

**Authors:** Jerzy Wojciechowski, Maciej Stolarski, Gerald Matthews

**Affiliations:** 1 Faculty of Psychology, University of Warsaw, Warsaw, Poland; 2 Institute for Simulation & Training, University of Central Florida, Orlando, Florida, United States of America; University of California, San Francisco, United States of America

## Abstract

Processing facial emotion, especially mismatches between facial and verbal messages, is believed to be important in the detection of deception. For example, emotional leakage may accompany lying. Individuals with superior emotion perception abilities may then be more adept in detecting deception by identifying mismatch between facial and verbal messages. Two personal factors that may predict such abilities are female gender and high emotional intelligence (EI). However, evidence on the role of gender and EI in detection of deception is mixed. A key issue is that the facial processing skills required to detect deception may not be the same as those required to identify facial emotion. To test this possibility, we developed a novel facial processing task, the FDT (Face Decoding Test) that requires detection of inconsistencies between facial and verbal cues to emotion. We hypothesized that gender and ability EI would be related to performance when cues were inconsistent. We also hypothesized that gender effects would be mediated by EI, because women tend to score as more emotionally intelligent on ability tests. Data were collected from 210 participants. Analyses of the FDT suggested that EI was correlated with superior face decoding in all conditions. We also confirmed the expected gender difference, the superiority of high EI individuals, and the mediation hypothesis. Also, EI was more strongly associated with facial decoding performance in women than in men, implying there may be gender differences in strategies for processing affective cues. It is concluded that integration of emotional and cognitive cues may be a core attribute of EI that contributes to the detection of deception.

## Introduction

Lying and deception are highly pervasive [1]. DePaulo et al.'s [2] classic diary study suggested that almost everybody lies at least once a week, and about 30% of lies regard feelings. People tell lies to pretend that they feel better than they do or to signal agreement with their partners. For successful deception, the verbal message should be coherent with nonverbal signals. Lewis [3] argues that emotional deception is part of ‘normal’ socialization (e.g., parents encourage their children to smile even if a gift was disappointing). Ekman and Friesen [4] pointed out that in order to deceive others her/his inner state, the liar can 1) simulate an emotional expression when s/he does not feel any emotion 2) mask emotion that s/he really feels with another emotional expression or 3) try to neutralize emotion s/he feels by showing neutral expression. However, fake emotional expression may be accompanied by emotional “leakage”. Even people adept at masking and simulating emotion cannot prevent leakage of real emotions [5]. The leakage of real emotions appears especially in the upper part of the face [6]. Emotional leakage has been demonstrated in studies of micro-expressions. According to Ekman [7], deception may be accompanied by a brief (<1/15 s) facial expression of emotion inconsistent with the speaker's statements. Speakers may have various motivations for concealing emotion. Such motivations are not necessarily deceptive, but deception may be one of the main contexts in which inconsistent microexpressions are expressed [8], [9].

Evidence from studies of microexpressions [8] implies that detection of microexpressions may contribute to competence in the detection of deception. The everyday lie may often be accompanied by a facial – verbal inconsistency. However, research need not focus solely on microexpressions. A recent, large-scale study [6] found that deceptive facial emotional expressions often lasted up to a full second, i.e., longer than microexpressions as defined by Ekman [7]. Furthermore, complete deceptive expressions were rare; partial microexpressions associated with only one part of the face were more common. Deceptive expressions were more common in the lower part of the face, perhaps because people have difficulty in voluntarily controlling the medial part of the frontalis muscle.

The present study thus focused on detection of inconsistency between relatively long duration facial emotion (2 s) and verbal content. There is rather little previous research on such inconsistencies. In the criminal justice system, it is often believed that the appropriateness of expressed emotion is important for evaluating the credibility of suspects and witnesses. For example, in the recent case of Amanda Knox, accused of murdering her friend in Perugia, Italy, her failure to express appropriate grief was one factor that led police to suspect her guilt [10]. Kaufmann et al. [11] showed in a simulation study that evaluations of the credibility of a rape victim's testimony were influenced by the extent to which she expressed socially-defined appropriate emotions such as despair. Another line of evidence comes from studies of depression. Clinical evidence suggests that depressed individuals may be adept at detecting false reassurances [12]. Dysphoric individuals are indeed more competent than controls in detecting lies made during videotaped statements, although they are also superior at detecting lies from voice alone [13].

### Individual differences in deception detection

Detecting lies requires paying attention to appropriate cues and interpreting them correctly. Nonetheless, studies showed that detection of deception among non-trained people as well as professionals only slightly exceeds the level of guessing (for review see: [14]). Indeed, knowledge about deception cues among both professionals (e.g., police officers) and lay persons is mostly incorrect [15]. Students have the same incorrect beliefs about the relevant cues indicating deception as customs officers, police detectives, police patrol officers and prison guards [16]. Apparently, prisoners have the most accurate knowledge about deception cues, because success in their world depends on their ability to detect deceit [16].

Although some researchers claim that it is unclear whether detection of deception is a stable characteristic [17] and meta-analysis lead to pessimistic conclusions [18], results of several studies suggest that people consistently vary in lie detection skills [19], [20]. Indeed, some researchers claim that ‘truth wizards’ – people who are particularly accurate in lie detection – really do exist [21], [22], [23]. It could be hypothesized that individuals exhibiting high emotional and social skills are better lie detectors. DePaulo and Tang [24] shown that observers low in social anxiety are better in deception detection than the ones with high scores. Deception detection is also positively correlated with self-awareness, which provides information about both one's own and someone else's mind [25]. However, extraversion, sociability and trust, which are as well socially valuable characteristics, are negatively correlated with discrimination between real and fabricated memories, while neuroticism facilitates effective lie detection [26], [27].

Analyses of gender differences also lead to inconsistent conclusions. On the one hand women are superior in detecting deception of their romantic partners [28]. This difference could be explained with their predominance in reading nonverbal cues (including facial expressions). Women are also superior in experimental ‘mind-reading’ tasks, i.e., inferring the thoughts and feelings of an acquaintance or partner from observing their behavior [29] and in perceptual sensitivity to very subtle non-verbal affective signals (e.g. positive facial expression) [30]. Females pay more attention to nonverbal cues and consider more of them during decision making [31], [32]. On the other hand, women's superiority vanishes in case of interaction with strangers [33].

Given that deception processes are highly emotionally loaded, gender differences in this area may result from more general sex differences in emotional processes. Gender differences in emotional experience, emotional expression, and nonverbal communication behaviors relating to emotion are among the most confirmed disparities between males and females [34]. Both differential socialization [35], [36] and evolutionary processes (e.g., [37]) may contribute to gender differences. Females have greater ability than males to perceive facial expressions of emotion as early as three years of age, but there may be various sociocultural moderator factors [38], [39], [40]. There may also be qualitative differences between the genders in which regions of the brain are activated during the perception of emotional expressions [41], [42]. Furthermore, literature reviews [43], as well as more recent studies (e.g., [44]), suggest a modest female advantage in accurate emotion recognition. Although some well-designed and substantial studies have failed to show any gender difference in facial emotion decoding [45], it is highly probable that some uncontrolled causes were responsible for the lack of gender effect (e.g., ceiling effect in the Hoffman et al.'s study [45]).

### How emotional intelligence can facilitate deception detection

Existing research has successfully sought for relatively reliable cues enabling effective deception detection (e.g., [46]), attempted to identify groups that perform better in lie detection (e.g., [21]), and investigated whether deception detection can be trained (e.g., [47]). However, it is unclear which individual difference variables would systematically enhance or weaken individual accuracy in judging deception [18]. In the present study, we examine whether emotional intelligence may prove crucial for individual effectiveness in detecting ‘emotional liars’.

Emotional intelligence (EI) has been one of the most often investigated, albeit controversial constructs, in contemporary psychology, since its introduction in 1990 by Peter Salovey and John Mayer [48] (see for a review: [49]). Development of reliable and valid measurement instruments has been especially problematic (e.g., [50]). Among numerous EI theories, the ability-based model developed by Mayer and Salovey [51] seems to have the strongest theoretical and empirical bases. Its strengths include its low redundancy with personality and IQ, and objective nature of EI measurement (i.e., maximum performance test). The MSCEIT test based on the model also appears to be a valid predictor of effectiveness in social and interpersonal activities [52], [53]. Therefore, we adopted the model as the conceptual basis for the present study.

Mayer and Salovey [51] distinguish four branches, each describing one group of emotional abilities: 1) perception, appraisal, and expression of emotion, 2) emotional facilitation of thinking, 3) understanding and analysing emotions, and employing emotional knowledge, and 4) reflective regulation of emotion. Each of the particular abilities constituting each branch may prove vital for detecting emotional deception.

First, an *ability to identify emotion* in other people (second ability of branch 1), which is often considered a core ability of EI [54], seem necessary (albeit not sufficient) for detecting emotional leakage and unmasking emotional liars. It seems obvious that without effective perception of emotion an individual is unable to detect an emotional deceit. Mayer and Salovey [51] explicitly describe an “*ability to discriminate between (…) honest dishonest expressions of feeling*” (p. 11) as a symptom of the highest level of branch 1 abilities. However, emotional perception is not the only ability necessary for detecting emotional lies. *Emotional facilitation of thought*, particularly an ability to use emotion to direct attention to important information, may support more basic emotional perception skills. *Emotional understanding* abilities, including recognizing relations between words and emotions themselves, help in interpreting the meaning that emotions convey regarding interpersonal interactions, as well as in recognizing likely transitions among emotions (see: [51]). Such emotional reasoning processes seem particularly important when one has to combine an interlocutor's verbal expressions with information coming from their facial expressions (such a “combined” strategy facilitates detecting deception [46]). Even the *emotional regulation* branch may prove useful in deception detection as it contains abilities “*to reflectively engage or detach from emotion depending on its judged informativeness and utility*” as well as to “*reflectively monitor emotions in relation to oneself and other*” ([51], p. 11).

Two studies have investigated EI in the context of deception. One revealed that individuals higher in the ability to perceive and express emotions feign emotions more convincingly than others, but they were still not immune to emotional leakage [5]. Similar results were reported by Elfenbein et al. [55]; however, in this study only emotion recognition ability, not overall EI, was measured. Both these studies investigated deception skills. The other relevant study [56], tested whether high EI was a major characteristic of ‘detection wizards’. Paradoxically, although total EI score was not related to discrimination of truths and lies, the perception branch score proved *negatively* related to detecting deceptive targets. However, the experiment design in this experiment was rather specific, engaging real-life videos of individuals emotionally pleading for the safe return of their missing family member, half of whom were responsible for the missing one's disappearance (or murder). Therefore, this study considered high-stakes emotional deception, and presented liars who could be characterized as psychopaths. Results may not generalize to the mundane lies of ‘everyday’ situations.

Perhaps as a result of gender differences in emotional-cognitive processes previously described in the previous section (see also: [57]), females are superior over males in EI, when the construct is considered as an ability and measured with a performance test (e.g., [58]). For self-reported measures the results are inconsistent, depending on the EI subscale (e.g., [59]). What is interesting, in some cases gender may moderate a relationship between EI and other variables (e.g., [60]). In the present study we used a performance-based measure of EI to investigate gender differences in detection of inconsistency in combined facial and verbal emotional signals.

### Aims and hypotheses

In the present study we aimed to determine whether EI predicts the ability to detect inconsistencies in emotional and verbal signals, using a novel facial-verbal decoding task for this purpose. Detection of such ‘mixed messages’ may contribute to lie detection. We hypothesized that (H1) higher EI is related to higher effectiveness in detection of inconsistency. Moreover, we presumed that (H2) females will score higher on an ability EI measure than males. These two hypotheses also imply that (H3) females should be more effective in detecting inconsistency than males. If EI is the critical factor in the gender difference, we also expect that (H4) the difference in detecting inconsistency may be statistically mediated by EI.

## Method

### Participants

210 research subjects (university students and community sample, Caucasians, 50% females) took part in the study (age range 18–53, *M = *23.7, *SD = *3.02). All subjects reported having normal or corrected to normal vision. They were naïve as to the purpose of the experiment. They were not rewarded.

### Ethic statement

All participants provided their informed consent to take part in the research prior to the experiment. The consent was obtained twice: a verbal one while participants were being invited to take part in the experiment and another one via the computer program. The second consent was obtained after detailed instruction. Participants were asked to write down their initials (recorded in a separate file in order to maintain anonymity) and press “next” button if they agreed to take part in the experiment. Otherwise they did not participate in the research. The study was approved by the Ethics Board at the Faculty of Psychology University of Warsaw. The participants were treated in accordance with ethical guidelines of Ethics Board of Faculty of Psychology University of Warsaw.

### Measures and stimuli

#### Emotional intelligence

EI was measured with TIE - the Emotional Intelligence Test [61]. This 24-item ability test was constructed on the basis of Mayer and Salovey's [51] four-factor model. The whole test consists of two parts with different instructions. Respondents are asked to read a series of descriptions of social interactions. In the first part, referring to Perception and Understanding, participants are asked to reflect on feelings and thoughts of persons who were involved in described situations. The task is to evaluate, on a 1–5 Likert scale, the probability that a person involved in the situation experiences each of them. In the second part, referring to Facilitation and Management, test-takers are asked to indicate the most advisable action that a protagonist should implement in order to solve the problem. The task is to judge, on a 1–5 Likert scale, the level of appropriateness of each of the three actions described on the answer sheet. Similarly to MSCEIT scoring [62] expert criteria were employed to determine the correctness of answers. The TIE responses are scored on four scales, consistent with Mayer and Salovey's [51] theory: Perception (Cronbach's α = .70), Understanding (α = .69), Assimilation (α = .65), Emotion Management (α = .66) and General Score (α = .88) [61]. TIE is a maximum performance test, intended to measure actual emotional abilities or “ability-based EI”. In terms of construct validity, the test has revealed a very similar pattern of relationships with established scales to the MSCEIT, correlating with fluid and crystallized intelligence (*r* = .35 and *r* = .26, respectively), with the strongest correlations for Understanding branch (similar to MSCEIT). TIE is generally independent from Big Five personality traits (only the dimension of Agreeableness revealed a significant relationship(*r* = .16), (similar to MSCEIT). As for convergent validity, TIE proved significantly correlated with SIE-T [63] (Polish test based on MSCEIT Faces subtest) and a Polish adaptation of Schutte et al.'s [64] Self-Report Inventory (SSRI). Stolarski, Bitner and Zimbardo [65] reported a correlation of .36 (*p*<.01) between TIE and the Popular Questionnaire of Emotional Intelligence [66].

#### Reasoning based on facial expressions

We consider lie detecting a complex cognitive-emotional task that requires comparing information emanating from multiple sources, e.g., facial expressions and verbal communications. Comparison of items of information is likely to be attentionally demanding, requiring use of working memory. The Face Decoding Test (FDT) is a specially designed computer test developed to measure individual effectiveness in reasoning based on facial expressions. The test consists of facial expressions, each followed by a sentence, both presented on a computer screen. Participants were asked to assess on 4 point scale whether the person who showed a particular emotional expression could honestly have said a presented sentence. The Vanger, Hoenlinger and Haken [67] computer generated prototypes of facial expressions of emotions were used in the FDT. These included facial expressions of basic emotions (joy, sadness, fear, disgust, anger, surprise), neutral facial expressions, as well as facial expressions composed of two inconsistent emotions – different for upper and lower facial muscles (e.g., the facial expression of a false/fake smile was composed of a neutral facial expression in the upper part of the face and joy in the lower part of the face). Presentation of these inconsistent facial expressions is pivotal for the whole idea of FDT, and for the present study. Emotional expression in the upper part of a face is less susceptible to intentional control, and, consequently, remains the more reliable indicator of truly experienced emotions [8], compared to the lower part, which is more easily controlled. Thus, we assumed that emotionally intelligent individuals will base their analysis in ambiguous situations mainly on the upper face.

Six facial expressions of basic emotions, neutral expression and eleven facial expressions of inconsistent facial emotions were used. Inconsistent facial expressions were selected on the basis of assessments by four competent judges – psychologists who have expertise in nonverbal communication of emotion, facial expression of emotion, or face processing. They were asked to indicate which emotions are most commonly masked, simulated or neutralized, and to indicate which facial expressions are used to hide them. Judges watched each inconsistent facial expression from Vanger, Hoenlinger and Haken's [67] study and evaluated it on three five-point scales: 1) commonness of the inconsistent facial expression in everyday life, 2) frequency of deceptive behavior connected with this inconsistent facial expression and 3) quality of computer generated prototype of the facial expression. Based on their evaluations, eleven inconsistent facial expressions rated highest on all three dimensions were chosen:

1. Indifference:
  - False smile – neutral facial expression (upper part) and joy (lower part).
  - False sadness – neutral facial expression (upper part) and sadness (lower part).
2. Joy:
  - Joy (upper part) and neutral facial expression (lower part).
  - Joy (upper part) and sadness (lower part).
3. Sadness:
  - Sadness (upper part) and neutral facial expression (lower part).
  - Sadness (upper part) and joy (lower part).
4. Fear:
  - Fear (upper part) and anger (lower part).
  - Fear (upper part) and neutral (lower part).
  - Fear (upper part) and joy (lower part).
5. Anger
  - Anger (upper part) and neutral (lower part).
  - Anger (upper part) and joy (lower part).

For each facial emotional expression six sentences were created. The same judges assessed each of them on two five-point scales: probability of appearance of the sentence in everyday life situations and correspondence of the sentence to a particular facial emotional expression. For each of the consistent emotional expressions the two sentences with the highest average judges' assessments were chosen: one that could be honestly said by a person on the just presented photograph, and a second which was inconsistent with the perceived expression (the person could not say it honestly). For example, for the expression of fear, two sentences were possible:

1. Consistent: Oh, my God! Watch out!
2. Inconsistent: It's really beautiful! I like spiders.

In total, 14 unique sets of facial expression and sentence for basic emotions and neutral face were created.

Also, for each of the inconsistent emotional expressions, the two sentences with the highest evaluations from the judges were chosen. Both sentences were written to reflect the emotion presented on the lower part of face (i.e., the “mask-emotion”; thus neither sentences could be said honestly). E.g., for the expression of fear masked with indifference:

1. Don't worry, I believe that everything is going to be alright.
2. It's not scary at all, I think you are overreacting.

The opposite combination, i.e., sentence consistent with the upper part of face, and inconsistent with the lower, were not used. Situations in which one tries to mask the truly experienced emotion and then formulate sentence consistent with it do not appear in interpersonal interactions. 22 unique sets of inconsistent emotional expressions and sentence were created.

In the FDT we decided to create two series of photo-sentence sets. In each series the same 36 photo-sentence sets were presented in different, pseudo-random order. The series were presented one after another without any pause. As a result each photo-sentence set was presented twice. We assumed that using multiple instances of each stimulus should improve reliability of the test.

**Figure 1:**
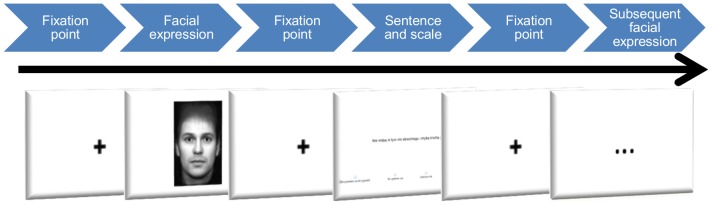
The structure of the example stimuli (facial expression) presentation in FDT (Face Decoding Test).

Stimuli were presented as follows (see Figure 1). Each presentation of facial expression lasted 2 s and was preceded with a fixation point presented in the middle of a screen. Next, a second fixation point was viewed for 1 s. Subsequently, a sentence was presented, accompanied by a four-point Likert-type scale to assess whether and to what degree a participant agrees that the statement could be honestly said by the person previously imaged. The response options were: 1 – definitely disagree, 2 – somewhat disagree, 3 – somewhat agree, 4 – definitely agree. The 4-point scale was applied instead of simple honest vs. dishonest decision, as we presumed that EI will influence not only detection of deception accuracy, but also the confidence of the answer. Afterwards, one more fixation point was presented, preceding presentation of the next trial. The program recorded participants' evaluation of each sentence as well as their reaction times. For each trial, the participant was awarded 1–4 points. On consistent trials, point allocations were as follows: definitely disagree  = 1, somewhat disagree  = 2, somewhat agree  = 3, definitely agree  = 4. On inconsistent trials, scoring was reversed, i.e., ‘definitely disagree’ was scored as 4, and ‘definitely agree’ as 1.

For each participant we obtained a total score in FDT and subscale scores for each condition: 1) basic emotions with congruent sentence, 2) basic emotions with non-congruent sentence, and 3) inconsistent emotions subscales. Moreover, the former two could be aggregated together as a 4) basic emotion condition. Each scale was scored by dividing the sum of points gained in all trials of a subscale by the number of trials. Thus, in each case the score ranges between 1 (the poorest performance possible) to 4 (the best possible performance). A value of 2.5 is expected by a chance.

### Procedure

Participants were tested individually. The Emotional Intelligence Test (TIE) was administered first, followed by FDT (Face Decoding Test. The whole procedure lasted about 40 minutes.

## Results

### Face decoding test (FDT) - Psychometric properties

The FDT scores rather closely approximates to a normal distribution, both in the case of total score (*M* = 2.78, *SD* = 0.25, skewness  = .53; kurtosis  = .77; Kolmogorov-Smirnov test value  = 0.85, *p* = .43) and for subscales: basic emotions scale (*M* = 3.17, *SD* = 0.33, skewness  = −.21; kurtosis  = −.51; Kolmogorov-Smirnov test value  = 0.89, *p* = .41); basic emotions with congruent sentences subscale (*M* = 3.22, *SD* = 0.38, skewness  = −.21; kurtosis  = −.34; Kolmogorov-Smirnov test value  = 1.09, *p* = .18); basic emotions with non-congruent sentences subscale (*M* = .13, *SD* = 0.41, skewness  = −.26; kurtosis  = −.13; Kolmogorov-Smirnov test value  = 1.18, *p* = .13); inconsistent emotions scale (*M* = 2.53, *SD* = 0.31, skewness  = .52; kurtosis  = .41; Kolmogorov-Smirnov test value  = 0.89, *p* = .41).

As anticipated, a one-way ANOVA showed that the differences between subscale means were significant, *F*(2,416) = 300.66, *p*<.001, partial η*^2^* = 0.59. Performance was highest for the basic emotions with congruent sentence subscale, lower for the basic emotions with non-congruent sentence subscale and the lowest for the inconsistent emotions scale. Post-hoc analyses (Scheffe tests) revealed significant differences between each subscale, all significant at *p*<.01 level.

The test revealed sufficient reliability, with Cronbach's alphas of .87 for total score (0,74 for first series and 0,78 for second series), .83 for basic emotions scale, .78 for basic emotions with congruent sentences subscale, .77 for basic emotions with non-congruent sentences subscale, .86 for inconsistent emotions scale. The correlation between first and second series amounted to *r* = .78, *p*<.001 for total score. This result confirms the FDT's reliability.

### Gender differences

A t-test for independent samples was conducted to compare results for men and women on TIE and FDT. The analyses showed that women (*M* = 27.97, *SD* = 5.12) scored significantly higher than men (*M* = 25.46, *SD* = 6.15) in TIE total score, *t*(201.34) = 3.22, *p*<.001, *d* = 0.45. Women obtained also higher results on all subscales of the emotional intelligence test (see table 1), with effect sizes ranging between .36 and .40. Therefore, the hypothesis H2 was confirmed.

**Table 1:** Descriptive statistics, mean comparisons, effect size estimations between females and males, N = 210.

	Females		Males				
	M	SD	M	SD	t	df	d
Emotional Intelligence							
Perception	7.81	1.62	7.09	2.05	2.83*	197.54	0.40
Understanding	7.37	1.62	6.73	1.83	2.69*	208	0.37
Assimilation	6.75	1.53	6.14	1.8	2.65*	202.66	0.37
Emotion Management	6,05	1.45	5.51	1.58	2.58*	208	0.36
Total score EI	27.97	5.12	25.46	6.15	3.22*	201,34	0.45
Face decoding test (FDT)							
Basic emotions	3.2	0.31	3.15	0.32	1.21	208	0.17
Basic emotions – congruent part	3.19	0.4	3.25	0.36	−1.13	208	0.16
Basic emotions – non-congruent part	3.21	0.39	3.04	0.41	3.1*	208	0.43
Inconsistent emotions	2.59	0.31	2.47	0.31	2.81*	208	0.39
Total Score FDT	2.83	0.26	2.74	0.24	2.74*	208	0.38

*Note.* The t-tests were two-tailed.

*p<.05.

**p<.001.

Also, in the FDT females (*M* = 2.83, *SD* = 0.26) scored higher than men (*M* = 2.74, *SD* = 0.24), *t*(208) = 2.74, *p* = .007, *d* = 0.38, which fully confirmed hypothesis H3. Further analyses revealed that this difference resulted mainly from females' advantage in detection of inconsistency, for the basic emotions with congruent communicates subscale was the only case in which the gender difference was not significant (see table 1).

### Relationships between EI and FDT performance

Further analyses were conducted to analyze a pattern of relationships between IE total score and branch scores and performance in the FDT test.

Analyses conducted on the whole sample revealed a systematic pattern of positive relationships between EI and all FDT measures, including emotional incongruence detection, with Pearson's *r* ranging between .20 and .38, all significant at *p*<.01 (see table 2), which fully confirmed hypothesis H1.

**Table 2:** Means, standard deviations and Pearson's correlation coefficients between TIE and FDT.

				Emotional Intelligence					Faces Decoding Test				
		M	SD	1.	2.	3.	4.	5.	I.	II.	III.	IV.	V
Emotional Inteligence	1. Perception	7.45	1.88										
	2. Understanding	7.05	1.76	0.64**									
	3. Assimilation	6.45	1.70	0.56**	0.64**								
	4.Emotion Management	5.78	1.54	0.53**	0.61**	0.69**							
	5. Total score EI	26.72	5.78	0.83**	0.86**	0.86**	0.82**						
Face Decoding Test	I. Basic emotions	3.17	0.33	0.37**	0.29**	0.34**	0.28**	0.39**					
	II. Basic emotions - congruent part	3.22	0.38	0.31**	0.21*	0.26**	0.20*	0.30**	0.83**				
	III. Basic emotions – non-congruent part	3.13	0.41	0.31**	0.28**	0.31**	0.27**	0.35**	0.85**	0.43**			
	IV. Inconsistent emotions	2.53	0.31	0.20*	0.18*	0.21*	0.25**	0.25**	0.22*	−0.08	0.42**		
	V. Total Score FDT	2.78	0.25	0.34**	0.28**	0.34**	0.33**	0.38**	0.68**	0.37**	0.76**	0.87**	

N = 210;

*p<.05;

**p<.001 (two-tailed).

Although the EI vs. FDT correlations were uniformly positive, there was some variation in magnitude. To test the extent to which the four TIE branches were differentiated as predictors of FDT, five multiple regressions were run. In each case, the four TIE branches were entered as predictors in a single step, and each of the five FDT measures, including total score, was treated as the criterion. All five equations were significant at *p*<.01, with R^2^ values ranging from .07 to .16. For all equations, except that with inconsistent emotions as the criterion, TIE perception branch score was the only significant predictor; βs ranged from .18 (*p* = .05) to .26 (*p*<.01). In the inconsistent emotions equation, no single predictor was significant. These analyses suggest that emotion perception may play the most important role in processing basic emotions, but the relationship between EI and processing of inconsistent emotional expressions is best attributed to EI as a whole, rather than any particular branch. Table 2 also shows that for all FDT subscales, except basic emotions – congruent, overall EI was at least as strongly correlated with performance as was emotion perception. Thus, in subsequent analyses we focus primarily on total EI as a predictor of the FDT.

It is worth mentioning that the FDT general score proved significantly negatively related to reaction time in the test, *r* = −0.14, *p*<.05. The result suggests that an “intuitive” strategy (i.e., characterized by rapid reactions) may be more effective than a “reflective”, strategy of deliberating over the response. However, further analyses showed that the negative correlation was significant only between reaction time and score on the basic emotions and congruent sentence subscale, *r* = −0.16, *p*<.05. This would rather suggest that the effect may simply reflect indecisiveness of individuals who were dealing poorly with this FDT condition. Moreover, no significant relationship between EI and FDT reaction time was obtained, both for the whole test and subscales.

Next, we repeated the correlation analyses separately for each gender, to test whether the criterion validity of EI generalized across both. This analysis (see table 3) revealed that the obtained relationships were particularly strong in women, with correlations ranging between .20 and .55 level. In men, correlation coefficients reached the .05 significance level only for basic emotions with congruent sentence, and only for perception and assimilation branches (i.e., for “experiential” EI as defined by Mayer, Salovey and Caruso [59]). To check the significance of the between-gender differences in strength of EI-FDT correlations, we performed series of comparisons of Pearson's r coefficients between genders using Steiger's Z. The differences proved significant for Basic emotions scale (especially for the non-congruent part) and for total FDT scores. In each of these cases the relationships were stronger in females, and in no case were they stronger in males. Thus, whereas in females EI appeared beneficial in all FDT conditions, in males emotional abilities facilitated performance only in the ‘congruent’ condition (i.e., when there was no “deception” to be detected).

**Table 3:** Means, standard deviations and Pearson's correlation coefficients between TIE and FDT.

					Males									
					Emotional Intelligence					Faces Decoding Test				
			M	SD	1.	2.	3.	4.	5.	I.	II.	III.	IV.	V
		M			7.09	6.73	6.14	5.51	25.46	3.15	3.25	3.04	2.47	2.73
		SD			2.05	1.83	1.80	1.58	6.15	0.32	0.36	0.41	0.31	0.24
Females	Emotional Intelligence	1. Perception	7.81	1.62		0.70**	0.61**	0.49**	0.85**	0.27*	0.27*	0.19	0.12	0.24*
		2. Understanding	7.37	1.62	0.53**		0.63**	0.61**	0.87**	0.13	0.15	0.06	0.05	0.10
		3. Assimilation	6.75	1.53	0.44**	0.64**		0.68**	0.86**	0.22*	0.24*	0.12	0.12	0.21*
		4.Emotion Management	6.05	1.45	0.55**	0.59**	0.68**		0.80**	0.12	0.15	0.06	0.16	0.19
		5. Total score EI	27.97	5.12	0.77**	0.84**	0.83**	0.84**		0.22*	0.24*	0.13	0.13	0.22*
	Faces Decoding Test	I. Basic emotions	3.2	0.35	0.47**	0.44**	0.46**	0.43**	0.55**		0.80**	0.85**	0.14	0.62**
		II. Basic emotions - congruent part	3.19	0.4	0.42**	0.31*	0.32*	0.30*	0.41**	0.88**		0.37**	−0.11	0.33*
		III. Basic emotions – non-congruent part	3.21	0.39	0.41**	0.46**	0.49**	0.45**	0.53**	0.87**	0.53**		0.31*	0.68**
		IV. Inconsistent emotions	2.59	0.31	0.22*	0.26*	0.26*	0.30*	0.31*	0.27*	−0.02	0.50**		0.86**
		V. Total Score FDT	2.83	0.26	0.41**	0.42**	0.43**	0.44**	0.52**	0.72**	0.44**	0.82**	0.87**	

N = 210; Females N = 105, Males N = 105;

*p<.05;

**p<.001 (one-tailed).

To investigate whether gender was a moderator of the relationship between EI and FDT performance, we performed interaction analyses, predicting each of the FDT scale scores with total EI score, gender and EI x gender interaction terms (centered). The models were significant in each case. The interaction terms were significant for all FDT dimensions, with an exception for the inconsistent emotions condition (see table 4).

**Table 4:** Testing significance of EI x Gender interaction terms for FDT total and subscale scores.

	Model statistics			Interaction term statistics		
Dependent variable	*F*(3,206)	*R^2^*	*p*	*β*	*ΔR^2^*	*p*
I. Basic emotions	16.26	.18	<.001	−.22	.05	<.001
II. Basic emotions – congruent part	9.86	.13	<.001	−.13.	.02	.047
III. Basic emotions – non congruent part	16.17	.18	<.001	−.23	.05	<.001
IV. Inconsistent emotions	7.01	.09	<.001	−.11	.01	.100
V. FDT total score	16.55	.18	<.001	−.19	.04	.003

**Figure 2:**
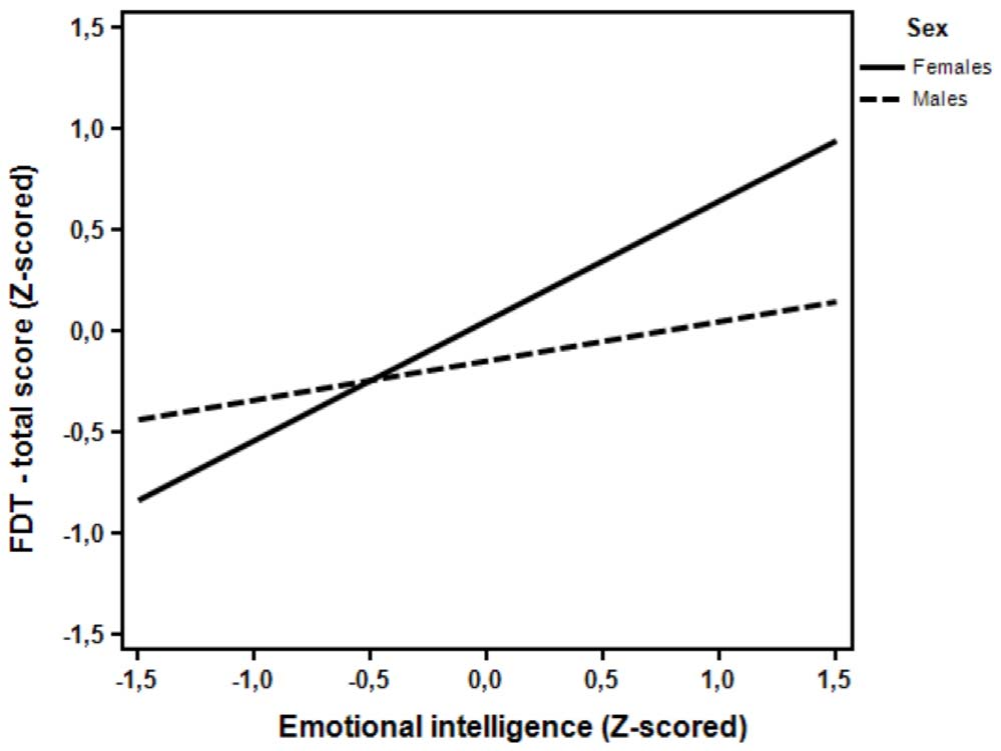
The relationship between EI and FDT score as moderated by gender.

To illustrate the obtained interactions we used Interaction! 1.4.1903 software by Daniel S. Soper [68], which allows for plotting graphical interpretation of the moderation effect. The effect was qualitatively similar for each of the FDT subscales and total score. Thus Figure 2 presents the interaction effect for total score, to illustrate the general form of the interactions.

**Figure 3:**
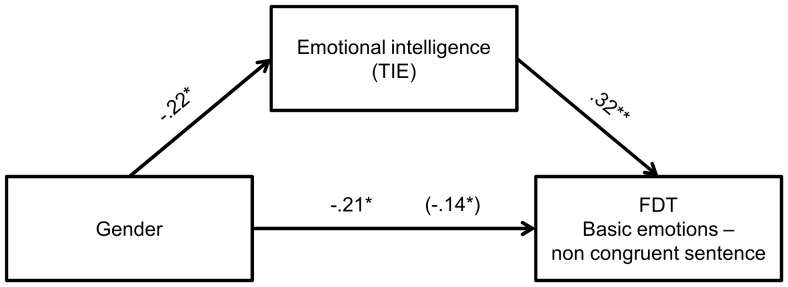
Relationship between gender and FDT basic emotions with non-congruent sentence as mediated by EI. The standardized regression coefficient between gender and deception detection controlling for EI is in parentheses. *p<.05, **p<.001.

**Figure 4:**
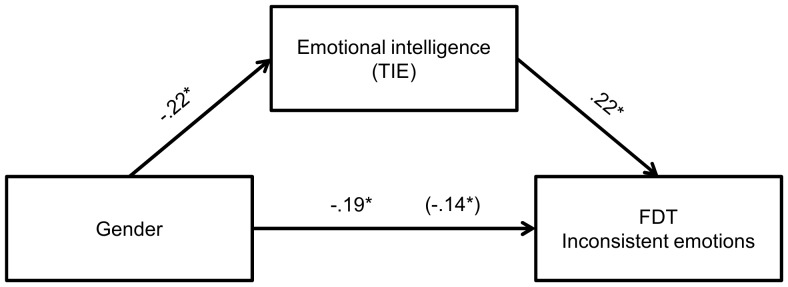
Relationship between gender and FDT inconsistent emotions subscale score as mediated by EI. The standardized regression coefficient between gender and deception detection controlling for EI is in parentheses. *p<.05.

**Figure 5:**
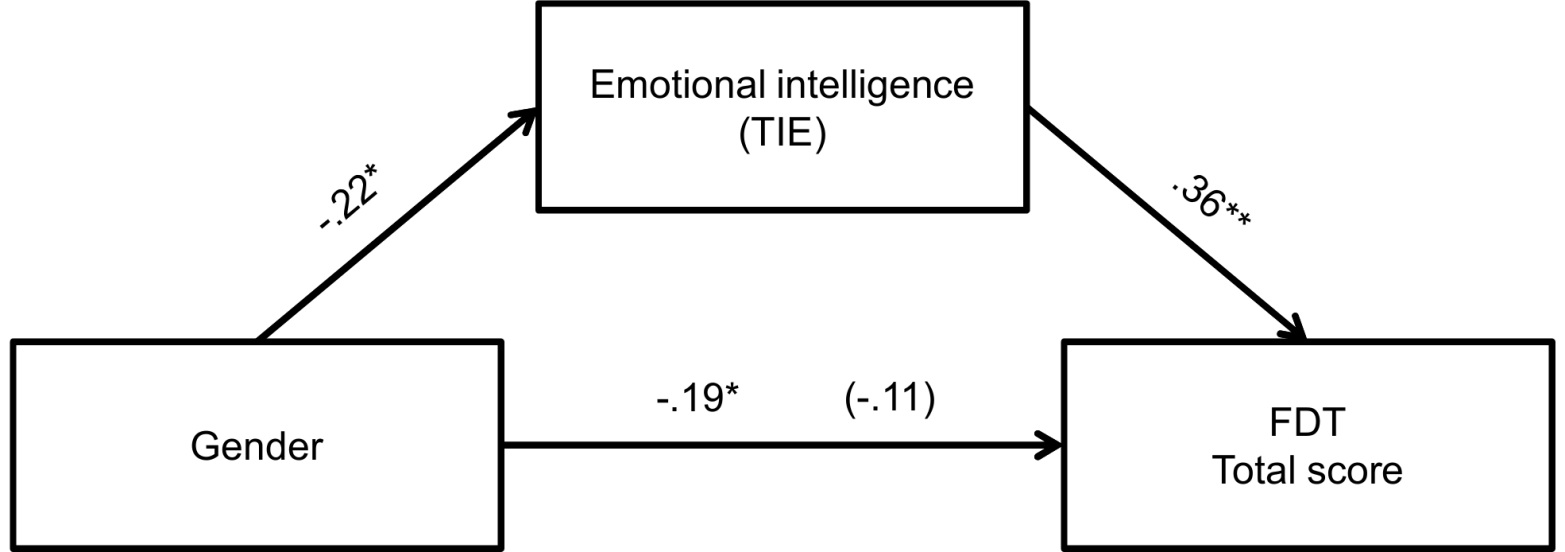
The relationship between gender and FDT total score as mediated by EI. The standardized regression coefficient between gender and deception detection controlling for EI is in parentheses. *p<.05, **p<.001.

Finally, we attempted to verify whether gender differences in EI could explain the gender difference in facial decoding. We therefore conducted mediation analyses (see figures 3, 4 and 5) for those FDT dimensions that revealed significant gender differences, i.e., basic emotions with non-congruent sentence, inconsistent emotions and total score. All three analyses revealed significant mediation effects, with Sobel test [69] values of −2.68, −2.25 and −2.78, respectively, all significant at p<.05 level (however for inconsistent and basic emotions scales it is only partial mediation).

Therefore, we may conclude that EI mediates between gender and effectiveness of deception detection. The H4 hypothesis was then also confirmed.

## Discussion

In this study, we investigated effects of EI and gender on performance on a novel facial emotion processing task (FDT), designed to simulate emotional deception detection. All formulated hypotheses were confirmed. More emotionally intelligent individuals performed better in all conditions of the FDT (H1). We also confirmed that females scored higher than males on both EI (H2) and FDT scores (H3). Interestingly, the gender difference on the FDT was significant only in the inconsistent FDT conditions. The mediation analysis confirmed H4, that gender differences in facial processing were mediated by EI. We also obtained two findings that were not hypothesized. The emotion perception branch of EI was the branch most predictive of performance when faces displayed basic emotions, but not when the facial emotion expression was inconsistent. We also found an intriguing gender difference in the correlational data; EI was more strongly associated with the FDT in women than in men, as confirmed by a test for moderation. In the remainder of this section, we discuss further the utility of the FDT in research on deception, and the roles of EI and gender in deception detection.

### Comments about FDT

The rationale for developing the FDT was that detecting inconsistencies between facial and verbal cues may be one strategy that people use to detect deception [46]. However, it could be arguable whether FDT indeed measures deception detection. Much prior research, inspired by Ekman's [70] seminal studies, has focused only on facial cues in isolation, e.g., detection of masked and simulated emotions [26]. Facial processing alone may indeed provide cues to deception. However, research reviewed by Barrett, Mesquita, and Gendron [71] shows that accurate emotion decoding relies on both processing of both facial expression and of concurrent contextual cues. Consistent with this principle, the FDT is not solely a facial processing task, but one which asks the respondent to evaluate verbal statements in the context provided by the facial emotion. Yet the experimental design endorsed by this authors was substantially different from FDT.

As a new task, findings from the FDT must be evaluated with caution. However, several features of the data suggest its utility as a research instrument. There were substantial overall differences in performance across conditions consistent with existing research. When the face stimulus displayed a basic emotion, mean performance was close to the upper end of the scale in both consistent and inconsistent sentence conditions. The near-ceiling levels of performance are consistent with the proposal that basic emotions are universal, and so emotion processing is highly accurate [4], [70]. The accuracy of processing extends not just to emotion recognition, but also to detection of inconsistency when the facial expression is unambiguous. By contrast, the mean score of 2.53 for inconsistent emotions expressions does not differ from the chance expectation of 2.5. The difficulty of detecting inconsistency under these conditions corresponds to findings that naïve participants typically do only a little better than chance in detection of deceptive facial emotion [8], [26].

Psychometric properties of the FDT are also encouraging. Distributions of scores in each condition were approximately normal, and acceptably internally consistent. Importantly, although there may be concerns about ceiling (basic emotion) and floor (inconsistent emotion) effects, reliable individual differences were obtained in each condition. In addition, the congruent basic emotions score was uncorrelated with the inconsistent emotions score, implying that the latter measures some ability more specific than either emotion recognition or sentence-image matching.

Some limitations of the FDT should also be noted. The face stimulus is artificial, in that prototypical facial expressions were created by averaging the photos/pictures of individual faces. Thus, the respondent views only a single ‘person’ expressing different emotions. However, using artificially generated facial expressions is quite common and accepted in contemporary cognitive science (e.g., [46], [72]). Furthermore, this individual was male, which may influence gender differences. Possibly, women have a special facility in reading male faces, although to date there is little evidence that congruence between participant gender and stimulus gender enhances recognition of emotion from facial expression [43], [73]. Finally, the stimulus presentation sequence separates the presentation of face and sentence, although naturalistically changing facial expression would accompany speech.

In future research, the FDT could be validated together with more established measures/paradigms of lie detection (for example a videotape study in which participants have to detect lies of the actors – e.g., [20], [74]). Apart from content and construct validation such a procedure could test whether the same or different skills are involved in both tasks. If results are promising, the FDT might eventually be used as a standardized assessment tool for deception detection.

### EI and deception

The data suggest a rather straightforward advantage for individuals high in EI, as assessed by the TIE. In the whole sample, all four TIE branch scores were significantly correlated with all three of the distinct FDT scores. Correlation magnitudes were similar, ranging from .18–31, although, as discussed below, there was rather more heterogeneity in correlations when males and females are examined separately. The aggregated total TIE and FDT scores showed a somewhat stronger association (*r* = .38) suggesting quite good criterion validity for the TIE as a predictor of detection of both consistency and inconsistency. EI appears to be advantageous irrespective of the difficulty of identifying inconsistency.

To the extent that the FDT taps processes that contribute to real-life detection of deception, the data suggest that emotionally intelligent individuals have an advantage in this respect in everyday interpersonal situations. Indeed, EI measured as an ability may contribute to range of social skills that support interpersonal functioning, as evidenced by several studies using the MSCEIT [75]. Evidence for validity of an ability measure of EI contrasts with the rather inconsistent evidence obtained in similar paradigms using questionnaire scales for ‘trait’ EI [76].

The present findings leave open the exact nature of the cognitive and/or emotional processes that may mediate EI – FDT association. The association might be attributed to the role of emotion perception as a foundational ability in the Mayer and Salovey [51] model. Perhaps, those high in EI simply identify facial emotion more accurately. In fact, the MSCEIT emotion perception branch has not been found to be a reliable predictor of performance on tasks requiring facial emotion processing [49]. For example, Roberts et al. [77] found no relationship between the MSCEIT emotion perception subtests and the JACBART test [78], which is based on Ekman's (e.g. [70], [79]) work. Here, we found that TIE emotion perception appeared from regression analyses to be the strongest predictor of performance in FDT conditions requiring decoding of basic emotion. By contrast with the MSCEIT, the TIE uses verbal instead of facial and graphical stimuli to assess emotion perception. Thus, the relationship between emotion perception and the FDT here reflects more than the common usage of facial stimuli in the two types of test. Importantly, though there was no special advantage for emotion perception in predicting performance in the inconsistent emotion condition of the FDT. In this condition, which may be the one most directly relevant to detection of deception, it may be general EI rather than any specific branch that is most predictive of performance.

We can advance two tentative explanations for the EI effect with inconsistent emotional stimuli. First, the task may be sufficiently complex to engage processes contributing to all four branches. Second, the integration of emotional (facial) and cognitive (linguistic) processes required to perform the task may represent a core function that is central to all aspects of EI. This hypothesis is compatible with Mayer and Salovey's [51] view that EI is a property of the linkages between emotion and cognition, rather than each domain in isolation.

### Gender differences

The advantage for females in ability EI is consistent with previous MSCEIT studies, which suggest a moderate effect size of around 0.5 SD (e.g., [60]), as here. Earlier studies conducted using TIE also revealed comparable effects (e.g., Stolarski, Postek and Śmieja reported a gender difference of .56 SD [80]). Effect sizes were similar across all four branches of the TIE. The findings from the FDT are also consistent with the general finding that females tend to perform better on tasks requiring decoding of nonverbal information, including facial expression. For example, in two meta-analyses, Hall [32], [43] found moderate effect sizes for the gender difference both for studies requiring identifying or interpreting nonverbal cues, and for the subset of studies investigating only visual cues, including facial cues. These nonverbal decoding cues may, in turn, contribute to the broader advantage in social skills reported for females (e.g., [81]). Collignon et al. [82] reported a particular advantage for women in recognizing multisensory emotional expressions, which they attributed to integration of vocal and facial expressions. The facility to integrate verbal and facial information is required for the FDT also. Our results are also in line with Hoffmann et al.'s [45] finding that gender differences are more pronounced for subtle expressions than for full-blown or high-intensity emotion displays.

The current findings add to existing knowledge by showing that gender differences are moderated by stimulus inconsistency. Males and females were equally adept at detecting consistency with basic emotion, but females were superior at detecting deception in both basic emotion and inconsistent emotions conditions. Effect sizes were similar to the value of *d* = .32 reported by Hall [43] for processing of visual cues. Multiple processes may contribute to the gender difference, but the effect here cannot be attributed either to a general facial processing advantage (which would imply female superiority in all conditions), or to an advantage in processing complex expressions (which would be restricted to the inconsistent condition). Women may be especially adept at processing inconsistent cues.

The mediation analysis suggested that female processing superiority might be attributed to higher EI. Consistent with our account of EI, we might suppose either that women are superior in a range of emotional competencies that jointly contribute to detecting inconsistency, or in some core process for EI of integrating cognitive and emotional information. However, there are two wrinkles in this simple account of gender differences that should be noted. First, high EI had an ‘across-the-board’ effect in enhancing all aspects of FDT performance, whereas gender differences depended on inconsistency. The mediation analysis may not pick up subtle differences between the advantages of high EI and the advantages of being female.

Second, the role of EI in the FDT seemed both stronger and less differentiated in women than in men. In the latter group, only the two ‘experiential’ branches of EI, perception and assimilation, predicted FDT. A tentative suggestion is that women are more motivated than men to rely on the explicit ‘strategic’ processes represented by the understanding and management branches, whereas men are reliant on more implicit, experiential processes. Consistent with this suggestion, gender differences in empathic accuracy (inferring the thoughts and feelings of another) appear to depend more on greater social motivation among women than on any basic ability [83]. Of course, over time, a greater interest in the feelings of others may contribute to building skills for emotion identification that may contribute to ability EI.

Tentatively, we suggest that our findings elucidate the superiority of women in detection of deception in some contexts [28]. In keeping with female superiority in decoding complex and subtle emotional stimuli [82], [45], women may also be better than men at detecting inconsistency between conflicting facial and verbal messages. This facility may be one of several competencies that contribute to detection of dishonesty in naturalistic settings. However, the use of artificial materials is a limitation of the current study, and caution is necessary in generalizing conclusions to real life deception.

## Conclusion

The basic findings from this study tell a simple story, that women are higher in men in general EI, and this ability helps women to identify inconsistent facial and verbal stimuli more readily. Such an ability might help women better detect emotional deception in real life, compared to men. The most parsimonious explanation for the performance advantage conferred by EI is that integration of emotional and cognitive information is a core attribute of EI, and one that is essential for detecting conflict between cues. However, there is also another interpretation of that effect. The relationship between EI and FDT could be a result of some common underlying ability. For example, both emotion perception and lie detection accuracy are considered part of “interpersonal sensitivity”, defined as accuracy in perceiving, judging, recalling, and responding to the (generally nonverbal) behavior and appearance of others [84], [85].

As a result, questions about the processing basis for the EI effect remain, including also whether multiple processes mediate effects on the FDT, the importance of motivation for performance, and the balance of strategic and experiential processes across the two genders. It also remains to be determined whether the FDT draws on those cognitive-emotional processes that support emotion deception in naturalistic settings.
